# Sustainability in the operating room: a cross-sectional survey of nurse anaesthetists’ and operating room nurses’ views and practice

**DOI:** 10.1186/s12912-025-03239-x

**Published:** 2025-05-26

**Authors:** Fiona M. Flynn, Susanne Skard Martinsen, Frida Øiseth, Jill Flo, Ann-Chatrin Linqvist Leonardsen

**Affiliations:** 1 Department of Nursing and Health Sciences, University of South-Eastern Norway, Campus Vestfold, Norway; 2 Østfold Hospital Trust, Halden, Norway; 3 Department of Nursing, Health, and Biomedical Sciences, Østfold University College, Halden, Norway

**Keywords:** Carbon footprint, Climate change, Drug residues, Nurse anaesthetist, Operating room nurse, Recycling, Survey, Waste reduction

## Abstract

**Background:**

The healthcare sector is responsible for between 4.4% and 5.2% of global greenhouse gas emissions, and the operating room accounts for 20–30% of hospital waste. Healthcare personnel have a responsibility to develop sustainable healthcare services that reduce waste and pollutants discharged into the environment. Nurse anaesthetists and operating room nurses can play a key role in ensuring sustainable practice in the operating room. The study’s aim was to (1) explore their views and practice regarding climate change, the healthcare sector’s carbon footprint, and sustainable practice in the operating room, (2) assess differences in the way in which the two professions regard these issues, and (3) assess factors which potentially are associated with participants’ views and practice.

**Methods:**

A cross-sectional survey was conducted in three hospital trusts in south-eastern Norway between October 2023 and January 2024. Responses were analysed using descriptive statistics, independent t-tests and Chi-square analyses. Thematic analysis was used for free-text responses.

**Results:**

A total of 110 nurse anaesthetists and 88 operating room nurses participated in the survey (response rate = 31%). Almost all participants (96.9%) agreed that the world is facing a climate change crisis. There were significant differences (*p* < 0.001) between the two professions’ views regarding the individual’s responsibility in reducing the healthcare sector’s carbon footprint. There was also a significant difference in their views regarding whether the health institution provided training to promote sustainable practice (*p* < 0.001). Both professions recycled waste materials, but there was a significant difference regarding the perceived need for recycling systems (*p* = 0.03). Three themes emerged from the free-text responses: ‘a *need for education and information’*, ‘*increasing awareness’* and ‘*lack of organisational measures’* regarding participants’ perspectives on climate, health and sustainability in the operating room.

**Conclusions:**

Despite certain differences in their views and a perceived lack of awareness, information and organisational measures, this study shows that nurse anaesthetists and operating room nurses are concerned about reducing the operating room’s carbon footprint. However, they have the potential to play a more active role in greening the operating room by participating in grassroot strategies that foster sustainability.

**Clinical trial number:**

Not applicable.

## Background

Climate change and global warming are regarded by the World Health Organization as the single greatest threat to human well-being and health in the 21st century [1]. Paradoxically, the healthcare sector is estimated to be responsible for between 4.4% and 5.2% of global greenhouse gas emissions and is a major contributor to consumption of scarce water resources, air pollution and nitrogen pollution of water [2–4]. Together this has an inadvertent negative effect on health, with a total negative environmental impact of 1–5% globally [3]. The supply chain for healthcare, which includes the production, transport and disposal of medical supplies such as pharmaceuticals, instruments and medical equipment, accounts indirectly for 71% of the healthcare sector’s emissions [2]. Operating rooms are major contributors to a hospital’s carbon footprint as surgery is particularly resource-intensive, requiring considerable amounts of energy, advanced technology and disposable equipment and materials, as well as generating substantial amounts of waste [5–8]. In addition, anaesthetic gases are potent greenhouse gases which are discharged directly into the environment, contributing to global warming [4, 9]. It is estimated that the operating room is directly responsible for as much as 20–30% of a hospital’s waste, with a quarter of this generated by anaesthesia [10, 11].

The Intergovernmental Panel on Climate Change (IPCC) has declared an urgent need for global action to reduce greenhouse gas emissions, and that time to secure a sustainable future for all is running out [12]. Thus, the healthcare sector has a responsibility to reduce its own carbon footprint and develop sustainable healthcare services that are resilient to climate change, conserve resources and reduce the amount of waste and pollutants discharged into the environment [13]. Recent research has shown growing concern among healthcare professionals regarding these issues, and various initiatives have been suggested to reduce the environmental impact of health services. These focus primarily on reducing energy consumption and waste, using renewable energy, reusing and reprocessing single-use equipment where possible, and segregating and recycling waste products [6, 14, 15]. There have also been initiatives to reduce the use of anaesthetic gases, particularly Desflurane, and develop anaesthetic gas capture technology [10, 16, 17]. Creating awareness, educating the workforce and developing guidelines are also regarded as important initiatives for promoting sustainable practice [6, 17].

In addition to their main responsibilities, nurse anaesthetists (NAs) and operating room (OR) nurses in Norway prepare any equipment and drugs to be used during the intraoperative phase and then dispose of waste materials and drug residues afterwards. OR nurses are responsible for infection control by ensuring the sterility of the procedure, which generates a considerable amount of waste material. Norwegian NAs independently provide anaesthesia to otherwise healthy patients, and in collaboration with anaesthesiologists to patients with more complex conditions [18]. Both NAs and OR nurses are postgraduate nurses with either a master’s degree (120 ECTS, European Credit Transfer and Accumulation System) or postgraduate diploma (90 ECTS) [19, 20]. Although their roles and responsibilities in the operating room differ, both professions are integral to ensuring that surgical procedures are carried out in an optimal and safe way. They have therefore the potential to play a key role in reducing the healthcare sector’s carbon footprint.

Recent studies on sustainability in the operating room have primarily focused on measuring the carbon footprint, establishing principles and strategies for environmentally-sustainable practices, in particular the reduction of anaesthetic gases, as well as attitudes and barriers towards implementation [6–8, 10, 17, 21, 22]. A previous study in Norway explored NAs’ and anaesthesiologists’ knowledge, views, and practice in relation to climate change, health, and sustainable anesthetic care [22]. However, this study focused predominantly on anaesthesia practice, and we have not identified any comparative studies exploring the views of NAs and OR nurses, two professions who are critical to ensuring that practice in the operating room is sustainable. Thus, the current study provides a novel contribution to understanding how this may be achieved.

## Methods

The aim of this study was to (1) explore NAs’ and OR nurses’ views and practice regarding climate change, the healthcare sector’s carbon footprint, and sustainable practice in the operating room, (2) assess differences in the way in which the two professions regard these issues, and (3) assess factors which potentially are associated with the participants’ views and practice.

### Design

A cross-sectional, observational design was used to explore NAs’ and OR nurses’ views and practice by means of a questionnaire. The study adhered to the STrengthening the Reporting of OBservational studies in Epidemiology (STROBE) guidelines [23].

### Study setting and sample

The study was conducted in eight hospitals belonging to three hospital trusts in southeastern Norway between October 2023 and January 2024. The study sample included all the NAs and OR nurses working a minimum of 50% of a full-time position in the operating department. Participants also had to be able to understand Norwegian well enough to respond to the questionnaire.

### Data collection

The questionnaire used in the study was originally developed to assess NAs’ and anaesthesiologists’ views and practice regarding climate change, healthcare and sustainability [22]. Minor modifications were made to the questionnaire with permission from Lindholm et al. to include the OR nurses and make it more specific to the nursing profession. The questions relating to disposal of pharmaceutical and single-use equipment and other waste were slightly modified to make them more appropriate for both professions. The modified questionnaire was tested in a pilot study by a group of student NAs (*n* = 3) and OR nurses (*n* = 4), and several minor adjustments were made to ensure it was comprehensible.

The first part of the final questionnaire included nine demographic questions, fifteen statements relating to participants’ views on climate change and the health care sector’s carbon footprint, and seven questions relating to sustainable practice in the operating room. A Likert scale ranging from 1 = strongly disagree to 5 = strongly agree, or 1 = to a very small extent to 5 = to a very large extent was used for questions about participants’ views. Four of the questions relating to sustainable practice allowed for multiple responses and free-text responses. Two final questions were added, allowing participants to write free-text answers about what could be done to increase recycling in operating rooms, as well as providing any additional comments.

An invitation to participate in the survey was emailed in October 2023 to the management of the operating and anaesthesia departments at all the participating hospitals, together with information about the study. The questionnaire was made available to participants via a link to a secure platform for data capture and storage, which also ensured complete anonymity. Two reminders were sent during the survey period, and the survey was closed in January 2024.

### Data analysis

Descriptive statistics were used to present the sample and proportions of the participants’ responses, reported as n (%). Independent t-tests with a Bonferroni correction to the *p*-value (*p* < 0.003) were used to study differences between the two professions. In the other tests a *p*-value < 0.05 was considered statistically significant. Chi-square analyses with Fisher’s exact test were used to study associations between categorical variables. Statistical analyses were performed using the IBM Statistical Package for the Social Sciences (SPSS), version 29.

The free-text answers were analysed using thematic analysis inspired by Braun and Clarke [24]. This method involved six steps. After familiarising themselves with the data (step 1), the authors inductively coded the free-text answers line by line identifying any content relevant to the study’s aims (step 2). Initial themes were identified and collated across the respondents (step 3). The themes were then discussed until agreement was reached between the authors (step 4). Step 5 included defining and naming the themes, and step 6 reporting the results.

## Results

The questionnaire was sent to 286 NAs and 335 OR nurses (*N* = 621). Of these, 110 NAs (38.5%) and 88 OR nurses (26.3%) responded, giving a total response rate of 31%. Two NAs and one OR nurse were removed from the sample, since they did not meet the inclusion criteria of working at least 50% of full-time employment. The characteristics of the participants are presented in Table 1.

**Table 1:** Characteristics of the sample (*N* = 195)

	*N*	%	Min.	Max.	Mean (SD)
Age in years	192		28	66	46.7 (9.9)
Gender Female Male	193 157 36	81.3 18.7			
Profession NA OR nurse	195 108 87	55.1 44.9			
Number of years as NA / OR nurse	194		1	44	12.9 (9.6)
Employment (percentage of full-time position) Full-time 80–90% Less than 80%	192 148 32 12	77.1 16.7 6.2			
Type of position Clinical Professional development Management Teaching Research Other	171 36 7 5 1 11	87.7 18.5 3.6 2.6 0.5 5.6			
Place of work Hospital Trust 1 (4 hospitals) Hospital Trust 2 (2 hospitals) Hospital Trust 3 (2 hospitals)	76 56 62	39.2 28.9 32.0			
Type of surgery in hospital Acute and elective Elective only	157 38	80.5 19.5			

NA = nurse anaesthetist. OR nurse = operating room nurse. SD = standard deviation

Table 1 shows that the participants were predominantly female (81.3%) and had worked as an NA or OR nurse for an average of 12.9 years. The professions were relatively evenly matched, 55.1% were NAs and 44.9% OR nurses.

### NAs’ and OR nurses’ views on climate change and the healthcare sector’s carbon footprint

Table 2 presents the NAs’ and OR nurses’ responses to the 15 statements regarding climate change and the healthcare sector’s carbon footprint, as well as differences between the two professions.

**Table 2:** NAs’ and OR nurses’ views on climate change and the healthcare sector’s carbon footprint (*N* = 195)

	Statement	Response *n* (%)						t-test
		Strongly disagree / disagree		Neither agree nor disagree		Agree/ strongly agree		*p-value*
		NA	OR nurse	NA	OR nurse	NA	OR nurse	
1	The world is facing a climate change crisis (*n* = 194)			2 (1.9)	4 (4.6)	106 (98.1)	83 (95.4)	0.28
2	I am worried about the consequences of climate change on public health (*n* = 194)	2 (1.9)	1 (1.1)	7 (6.5)	8 (9.2)	99 (91.7)	78 (89.7)	0.80
3	Hospitals should limit their impact on climate and the environment (*n* = 194)		7 (8)	1 (0.9)	4 (4.6)	106 (99.1)	76 (87.4)	< 0.001*
4	The healthcare sector has such a low carbon footprint that environmentally friendly changes will not make any difference (*n* = 194)	99 (91.7)	71 (82.6)	8 (7.4)	12 (14)	1 (0.9)	3 (3.4)	0.05
5	Reducing the carbon footprint is the responsibility of the health institution or its management (*n* = 194)	6 (5.6)	1 (1.1)	8 (7.4)	6 (7)	94 (87)	79 (91.9)	0.15
6	Reducing the carbon footprint is the responsibility of the individual health professional (*n* = 194)	9 (8.3)	14 (16.1)	12 (11.1)	28 (32.2)	87 (80.6)	45 (51.7)	< 0.001*
7	The healthcare sector plays an important role in responding to crises that impact on health (*n* = 194)		1 (1.1)	7 (6.5)	11 (12.6)	100 (93.5)	75 (86.2)	0.07
8	Nurses and doctors have a particular responsibility to warn against health threats (*n* = 194)	4 (3.7)	2 (2.3)	11 (10.2)	14 (16.3)	93 (86.1)	70 (81.4)	0.62
9	A «green» healthcare sector is a more attractive place to work (*n* = 194)	1 (0.9)	2 (2.3)	26 (24.3)	22 (25.9)	80 (74.8)	61 (71.8)	0.53
10	The healthcare sector should lead by example in preventing health loss caused by climate and environmental impact (*n* = 194)	3 (2.8)	2 (2.3)	3 (2.8)	6 (6.9)	101 (94.4)	79 (90.8)	0.57
11	The healthcare sector has too many other concerns to allow it to emphasize environmental concerns (*n* = 194)	88 (81.5)	49 (56.3)	9 (8.3)	22 (25.3)	11 (10.2)	14 (16.5)	0.003*
12	NAs have an individual responsibility to limit the impact of climate and environmental factors (*n* = 194)		8 (9.2)	13 (12)	25 (28.7)	95 (88)	54 (62.1)	< 0.001*
13	OR nurses have an individual responsibility to limit the impact of climate and environmental factors (*n* = 194)		8 (9.3)	13 (12)	25 (29.1)	95 (88)	53 (61.6)	< 0.001*
14	Surgeons have an individual responsibility to limit the impact of climate and environmental factors (*n* = 194)		8 (9.3)	15 (13.9)	27 (31.4)	93 (86.1)	51 (59.3)	< 0.001*
15	Anaesthesiologists have an individual responsibility to limit the impact of climate and environmental factors (*n* = 194)		8 (9.3)	12 (11.1)	26 (30.2)	93 (88.6)	52 (60.5)	< 0.001*

NA = Nurse Anaesthetist. OR = Operating room nurse. Independent t-test: *p < 0.003 = statistical significance

Table 2 shows that almost all participants (96.9%) agreed or strongly agreed that “the world is facing a climate change crisis”, and the majority (84%) agreed or strongly agreed that “nurses and doctors have a particular responsibility to warn against health threats”. They also agreed or strongly agreed that the healthcare sector “plays an important role in responding to crises that impact on health” (90.2%), “should lead by example in preventing health loss caused by climate and environmental impact” (92.8%), and that “a green healthcare sector is a more attractive place to work” (73.4%). Although the majority of participants (89.2%) agreed or strongly agreed that the health institution or its management had a responsibility to reduce the carbon footprint, there were statistically significant differences between the professions regarding their views on individual responsibility towards reducing the carbon footprint (*p* < 0.001). A significantly higher proportion of NAs responded that hospitals should limit their environmental impact (*p* < 0.001).

### Sustainable practice in the operating room

The survey included seven questions regarding sustainable practice in the operating room. The first two questions were concerned with the extent to which participants thought that measures had been implemented to promote sustainable practice through training and by following guidelines. The results are presented in Table 3.

**Table 3:** NAs’ and OR nurses’ views on whether measures were implemented to promote sustainable practice

		Response *n* (%)						t-test
		Small/ very small extent		Some extent		Large/ very large extent		*p-value*
		NA	OR nurse	NA	OR nurse	NA	OR nurse	
1	Training (*n* = 193)	35 (33)	43 (49.4)	49 (46.2)	40 (46)	22 (20.8)	4 (4.6)	< 0.001*
2	Guidelines (*n* = 195)	39 (36.1)	37 (42.5)	45 (41.7)	40 (46)	24 (22.2)	10 (11.5)	0.10

NA = Nurse Anaesthetist. OR = Operating room nurse. Independent t-test: *p* < 0.003 = statistical significance

Table 3 shows a significant difference in the extent to which the professions reported that training was provided to promote sustainable clinical practice.

The participants were also asked about recycling of waste materials, what happened to unused single-use consumables that had been opened, and how pharmaceutical waste was disposed of after surgical procedures. The responses to these three questions and a further question regarding what kind of measures were needed to reduce the amount of waste in the operating room and increase sustainability, are presented in Table 4. Multiple responses were allowed for these four questions.

**Table 4:** Sustainable practices and measures needed to reduce waste and increase sustainability

Question	Response *n* (%)			Chi-square
	NA	OR nurse	Total	*p*-value
If your department recycles, what is recycled?				
- Plastic - Paper - Glass and metal - Batteries - Electronic equipment - Other	84 (43.1) 94 (48.2) 44 (22.6) 84 (43.1) 49 (25.1) 5 (2.6)	67 (34.4) 70 (35.9) 35 (17.9) 78 (40) 39 (20) 0	151 (77.4) 164 (84.1) 79 (40.5) 162 (83.1) 88 (45.1) 5 (2.6)	1.0 0.24 1.0 0.03* 1.0 0.07*
What happens to single-use consumables that are opened but not used?				
- Thrown in general waste containers - Reused professionally - Reused privately - Recycled - Do not know - Other	70 (35.9) 45 (23.1) - 14 (13) 17 (15.7) 18 (9.2)	73 (37.4) 44 (22.6) 33 (37.9) - - 6 (3.1)	143 (73.3) 89 (45.6) 33 (37.9) 14 (13) 17 (15.7) 24 (12.3)	0.003* 0.25 - - - 0.05*
How is pharmaceutical waste disposed of after surgical procedures?				
- Prescribed container, returned to pharmacy - General waste containers - Washed down sink - Hazardous waste (yellow containers) - Do not know	57 (29.2) 44 (22.6) 6 (3.1) 73 (37.4) 1 (0.5)	36 (18.5) 24 (12.3) 3 (1.5) 62 (31.8) 2 (1)	93 (47.7) 68 (34.9) 9 (4.6) 135 (69.2) 3 (1.5)	0.15 0.07 0.73 0.64 0.59
What measures are needed to reduce the amount of waste and increase recycling in the OR?				
- Time for training - Money for training - More accessible recycling systems - Management directions, guidelines, local procedures - Choosing equipment suppliers with low carbon footprints - Other	23 (11.8) 6 (3.1) 96 (49.2) 69 (35.4) 61 (31.3) 4 (2.1)	20 (10.3) 6 (3.1) 67 (34.4) 50 (25.6) 59 (25.6) 5 (2.6)	43 (22.1) 12 (6.2) 163 (83.6) 119 (61) 111 (56.9) 9 (4.6)	0.86 0.77 0.03* 0.38 1.0 0.52

NA = Nurse Anaesthetist. OR = Operating room nurse. Chi-square analysis with Fisher’s exact test: *p < 0.05 = statistical significance

The question regarding single-use consumables included some different alternatives for the two groups and it was therefore not possible to analyse differences between the professions for all the responses.

No significant difference was found in the way in which NAs and OR nurses recycled waste, except that OR nurses recycled a significantly higher number of batteries (Fisher’s Exact test *p* = 0.03). In addition, OR nurses were significantly more likely to throw unused single-use consumables in general waste containers (Fisher’s Exact test *p* = 0.003). There was no significant difference in the way in which the two professions disposed of pharmaceutical waste. A total of 47.7% of participants disposed of drug residues correctly in prescribed containers for returning pharmaceutical waste to the pharmacy or in hazardous waste containers (69.2%). There were no statistical differences between the professions’ views on what kind of measures were needed to reduce waste, except regarding the need for accessible recycling systems. The NAs were significantly more concerned about the accessibility of recycling systems (Fisher’s Exact test *p* = 0.03).

The final question on sustainable practice concerned the extent to which the participants used the available recycling systems. The majority (81.5%) used them to a large or very large extent, 14.8% used them to some extent and only 3.7% used them to a small or very small extent, with no statistical difference between the professions. A chi square analysis showed that the type of surgery (emergency/elective) had no impact on their use of recycling systems.

### Free-text comments

A total of 30 NAs and 32 OR nurses provided suggestions on how to increase sustainability in the operating room. A common theme for both professions was a *‘need for education and information’*. Both groups reported that sustainability was not included as part of their education. Furthermore, they lacked information about how to recycle, and there were no guidelines or procedures in place. They regarded these measures as essential for ensuring sustainability. Another common theme was *‘increasing awareness’*. Both the NAs and OR nurses reported that they lacked awareness about making their own practice sustainable, and the NAs also reported that a team approach (including cleaning personnel) was lacking to increase awareness.

In total, 14 NAs and 16 OR nurses added comments regarding their perspectives on climate, health, and sustainable practice in the operating room. A common theme was the *‘lack of organisational measures’*. Both the NAs and OR nurses reported that a lack of waste disposal and recycling containers made it difficult to sort waste correctly. For example, paper was thrown in the plastic disposal container. However, the NAs regarded recycling measures as a management responsibility, rather than the responsibility of the individual.

## Discussion

This study explored NAs’ and OR nurses’ views regarding climate change, the healthcare sector’s carbon footprint and sustainable practice in Norwegian operating rooms. Although both professions agreed on a number of issues, for example, that the world is facing a climate change crisis and they were concerned about the effects on public health, there were also significant differences between their views on several central issues. A significantly higher proportion of NAs agreed that they had an individual responsibility to reduce the health sector’s carbon footprint, that hospitals should limit their environmental impact and disagreed that hospitals had too many other concerns to focus on, compared with the OR nurses. Although there were some significant differences, both professions recycled a variety of waste materials, and used prescribed containers for disposing of pharmaceutical waste some of the time. Both professions reported a need for information and education to increase awareness, as well as organisational measures that enabled sustainable practice.

The results in this study regarding the climate crisis and its negative impact on public health are supported by findings from anaesthesia personnel in another Norwegian survey [22]. The proportion of OR nurses (51.7%) who recognized the responsibility of individual health professionals in reducing the carbon footprint was comparable to anaesthesia personnel (57%) in Lindholm et al.’s study [22]. However, individual responsibility was recognized by a significantly higher proportion of NAs (80.6%) in the current study, similar to a German survey where 88.5% of anesthetists recognized an ethical responsibility to mitigate climate change [25]. Moreover, a significantly higher percentage of NAs than OR nurses in the current study also agreed that NAs, OR nurses, surgeons and anaesthesiologists have an individual responsibility to limit environmental impact. It is difficult to ascertain the cause of the differences between the views of the NAs and OR nurses, but it may be related to dissimilarities in their roles and responsibilities. Both professions are bound by regulations governing their professional practice to promote sustainability in their work [26, 27]. However, while NAs in Norway have an individual responsibility for their patients when administering anaesthesia [18], OR nurses work in close collaboration with other members of the surgical team and may feel more of a collective rather than a personal responsibility.

Most of the NAs (87%) and OR nurses (92%) agreed that reducing the carbon footprint is the responsibility of the health institution, a slightly higher number than in a survey among anaesthesia personnel (84.2%) [22]. However, they differed in their opinions about the extent to which training and guideline recommendations to promote sustainable practice were implemented. Both professions commented on a lack of education and awareness about how to make their practice more sustainable, as well as a lack of sustainability commitment among management and colleagues. An Australian review [7] identified a lack of management leadership policies and poor implementation of guidelines as barriers to fostering a sustainability culture, while another study reported that although anaesthetists believed their organisation was capable of implementing sustainable measures, there was a lack of cultural readiness [25]. This involved for example, the employer encouraging sustainable practice and defining targets for reducing emissions. Further barriers to sustainability were inadequate training and information [28] and behavioural factors such as engrained attitudes and resistance to change among staff [7]. Since the operating department is a clearly defined part of the hospital with its own supply chain, strategies to reduce its environmental impact can potentially be highly effective if all the professional groups working there are committed to changing behavioural patterns and making improvements [8]. There is therefore a need for hospital management to take the lead by providing education and information about the health sector’s environmental impact and formulating policies and guidelines to implement sustainability measures that staff can follow [6, 7].

The two professions differed significantly in whether they believed that the healthcare sector had too many other concerns to focus on environmental issues. A total of 81.5% of NAs disagreed with this, compared with only 56.3% of the OR nurses. In a similar survey, 71.1% of anaesthesia personnel disagreed that this was the case [22]. OR nurses are very aware of the volume of waste generated in the operating room resulting from infection control and the absolute need for sterility in surgical procedures. Surgical equipment often has several layers of sterile packaging, and there is a high consumption of single-use consumables, such as masks, gloves, gowns, surgical drapes, sharps, airway devices, intravenous lines, glass ampoules and syringes [7]. In addition, equipment is often opened and drugs prepared that are not used and must be disposed of, accounting for up to 30% of the waste generated during surgical procedures [7, 17, 29]. The OR nurses’ views may therefore be associated with their commitment to ensuring adequate infection control for the patient, which is a major part of their role [19].

The participants in this study showed a great willingness to recycle (81.5%), using the available recycling systems to a large extent in order to recycle paper, plastic, glass and metal and electrical equipment. However, they stated that appropriate recycling containers should be more accessible, and there was a need for guidelines to ensure waste was segregated correctly. Similarly, an Italian study found that 57% of waste materials were disposed of incorrectly, and 71% of these could have been recycled [5]. Several other studies had comparable findings, where large amounts of recyclable or hazardous waste were thrown in general waste containers [15, 30]. Incorrect sorting is often due to personnel being unable to distinguish between materials, inadequate labelling, inaccessible containers, or a lack of information about how to dispose of them correctly [15, 31]. Various studies have also identified staff attitudes, logistical problems and lack of time as potential barriers to optimal waste management [7, 21, 31].

Single-use consumables in the operating room are a major carbon hotspot [11, 32], and 73.3% of the participants in this study admitted that single-use equipment that was opened and unused was disposed of in the general waste. The perceived infection risk is a major barrier to reusing equipment, and there is a need for better education [7]. It is both feasible and effective to reprocess disposable medical devices but remains illegal in many countries, including Norway [6, 33]. A recent review pointed out that customized surgical procedure packs and technologically advanced procedures, in particular those using robotic approaches, are considerably more environmentally polluting than traditional approaches, while reuse of laryngoscope blades, laryngeal mask airways, surgical gowns and drapes, and surgical instruments among other things could reduce emissions [34].

Although participants generally disposed of drugs in prescribed or hazardous waste containers, 34.9% admitted to throwing drug residues in the general waste or washing them down the sink (4.6%). The participants could give multiple responses and apparently mostly followed the correct procedures, but sometimes for unspecified reasons did not. The consensus guidelines for environmentally sustainable anaesthesia recommend that pharmaceuticals should be disposed of in an environmentally sustainable manner [17]. Additionally, pre-filled syringes and infusion bags prepared by the in-house pharmacy or bought from a supplier, can reduce unnecessary pharmaceutical waste and pollution while simultaneously increasing a drug’s shelf-life, and are recommended for use in both routine and emergency situations [11].

### Clinical implications

NAs and OR nurses can play an active role in reducing environmental impact by encouraging behavioural change in several ways:

- by increasing awareness among colleagues and other staff in the operating room through the use of prompts and cues to remind staff to turn off machines, dispose of waste materials and drug residues correctly as well as discussing sustainability initiatives in departmental meetings.
- by reducing the amount of equipment and drugs that are prepared in advance and the use of anaesthetic gases to a minimum of what is absolutely necessary without jeopardizing patient safety or comfort.
- by providing feedback about surplus materials and waste to product suppliers, surgeons and other decision-makers and playing an active role in product selection to ensure that sustainable choices are made.
- by participating in research and professional development projects and devising innovative strategies for implementing sustainable practice solutions in the operating room.

### Limitations

This study used a convenience sample from one area in Norway and had a relatively low response rate. These factors together with variations in scope of professional practice internationally, limit the generalisability of findings. However, the sample consisted of both NAs and OR nurses and represented a variation in age, gender and experience, therefore the findings may be transferable to other settings. The questionnaire used in the survey was tested among anaesthesia personnel in Norway, and it is possible that it was not equally applicable to OR nurses, even though it was tested in a pilot study after adjustments were made. The analysis of free text responses did not achieve saturation therefore a higher number of responses may have provided other results. This underlines the need for qualitative approaches to gain in-depth insight into participants’ views on sustainability. Attempts were made to ensure transparency throughout the research process to increase the validity of the findings.

## Conclusions

Despite certain differences in their views and a perceived lack of awareness, information and organisational measures, this study shows that NAs and OR nurses are concerned about reducing the operating room’s carbon footprint. However, there is great potential for improvement. NAs and OR nurses can play a more active role in greening the operating room by participating in grassroot strategies that foster sustainability. These include working together to raise awareness, reduce waste and ensure that it is segregated and disposed of correctly; participating in product selection to ensure sustainable choices; and applying pressure on decision-makers in the health sector to provide organisational measures, such as guidelines and training. There is a need for further research into ways of overcoming barriers to sustainable practice.

## Data Availability

Anonymized data supporting the conclusions of this article will be made available by the corresponding author upon reasonable request.

## Abbreviations

NA: Nurse Anaesthetist
OR: Operating room nurse
ECTS: European credit transfer and accumulation system
SD: Standard deviation
